# A perfectionism, mental health and vulnerability to injury in triathletes

**DOI:** 10.3389/fpsyg.2025.1561432

**Published:** 2025-04-22

**Authors:** Laura Gil-Caselles, Roberto Ruiz Barquín, José María Gimenez-Egido, Alejo García-Naveira, Aurelio Olmedilla

**Affiliations:** ^1^Research Group HUMSE, Faculty of Sports Sciences, University of Murcia, Murcia, Spain; ^2^Interfaculty Department of Developmental and Educational Psychology, Autonomous University of Madrid, Madrid, Spain; ^3^Department of Psychology, University Villanueva, Madrid, Spain; ^4^Research Group HUMSE, Faculty of Psychology, University of Murcia, Murcia, Spain

**Keywords:** mental health, triathlon, perfectionism, injuries, moods

## Abstract

**Background:**

Triathlon is a highly demanding and continuously growing sport in continuous growth. Due to its complexity, it often exposes the triathlete to a mental and physical load that is difficult to manage and exposes the athlete to sporting injury. Many triathletes are increasingly developing problems related to mental health and because of this, a greater vulnerability to suffer a sports injury is being observed.

**Methods:**

The study sample included 172 triathletes (66.27% men, 33.72% women) categorized as Amateur, Youth, or Elite. Mean age: Amateur 40.19 ± 8.35, Youth 19.50 ± 2.63, Elite 30.38 ± 8.77. Weekly training: Amateur 5.63 ± 1.07 h, Youth 5.52 ± 1.28 h, Elite 6.58 ± 0.67 h. Daily training: Amateur 2.16 ± 1.66 h, Youth 2.38 ± 2.12 h, Elite 3.02 ± 2.00 h. Data collection involved administering a questionnaire assessing personal and sports-related variables, injury history, and mental health indicators. The psychological assessment included the Multidimensional Perfectionism Scale (MPS), the Depression, Anxiety, and Stress Scales (DASS-21), and the Profile of Mood States (POMS). The study design is descriptive-cross-sectional and has a retrospective character.

**Results:**

Significant positive correlations were found between TNL and TGSP across participants level, with the strongest correlation observed among youth athletes. Maladaptive perfectionism exhibited a weak negative correlation with TGSP in amateur athletes. Additionally, depression and stress were positively associated with TNL and TGSP, particularly in elite and youth athletes. Emotional state analysis showed that tension and depression were positively correlated with TNL and TGSP, whereas vigor was negatively correlated in elite athletes.

**Conclusion:**

These findings suggest a significant relationship between mental health and sports injuries, indicating that psychological distress not only contributes to injury risk but may also prolong recovery time in triathletes.

## Introduction

One of the sports that has seen the greatest growth in recent years is triathlon (Arenillas and Ruiz-Barquín, 2023; Hernández et al., 2014; Walker et al., 2014), made up of different disciplines: swimming, cycling and running (Cejuela et al., 2007; Li et al., 2025; Zhao et al., 2024). Currently, this sport is attracting a large number of practitioners, and although it had not attracted much attention from researchers, it is now on the rise (López-Cazorla et al., 2015; Weir et al., 2025; Zapata et al., 2023).

Triathlon is a multisport (Guevara et al., 2024; Puccinelli et al., 2024; Rimmer and Coniglione, 2012) individual and mainly aerobic endurance sport that involves a physical activity that involves different muscle groups of the human body, in addition this particularity makes it a very complete sport, since triathlon is a very demanding test that often exposes the athlete to a wide range of injuries (Bales and Bales, 2012a; Gil-Caselles et al., 2023; Laird and Johnson, 2012; Vleck and Garbutt, 1998) that can affect them during the practice of its three disciplines: swimming (Bales and Bales, 2012b), cycling (Deakon, 2012), and running (Spiker et al., 2012).

On the other hand, the high training volumes in endurance sports and, in the case of triathlon, the simultaneous training of three different sports disciplines must be controlled, as they require careful planning due to the large number of training sessions each week (Millet et al., 2011; Mujika, 2014; Tønnessen et al., 2024). Nowadays, it is well known that psychological variables are significant aspects in sports performance, both in elite and amateur athletes (Abdullah et al., 2016; Castilla and Ramos, 2012; Raimundi et al., 2022; Macnamara et al., 2010; Swann et al., 2017), therefore, it is not only necessary to take into account the physical load during training and competition, but it is also vital to know how to manage the mental load in a sport as complete and complex as triathlon.

One of the problems faced by the triathlete is sports injuries. The incidence of injuries varies according to age, sex, duration, and type of training. The level of practice also influences the risk of injury, being higher in elite triathletes. This sport is currently classified as one of the most demanding multidisciplinary sports (Etxebarria et al., 2019; Zhao et al., 2024) and its practice has been observed to generate great stress, since, on the same day and during the same competition, three consecutive activities must be performed, which increases its complexity and consequently can increase the incidence of diseases and injuries if they do not work properly, however, recent advances in knowledge in this area can minimize this risk and maximize performance. Some authors have defined triathlon-induced injury as: any bodily harm, in this case caused by triathlon training or competition, due to which, for at least 1 day, training has been stopped, training intensity/load has been lowered, medication has been taken or medical help has been sought (Burns et al., 2003; Collins et al., 1989; Mawlichanów et al., 2024; Qu et al., 2025; Vleck and Garbutt, 1998).

Several studies (Burns et al., 2003; Collins et al., 1989; Garós et al., 2024; Migliorini, 2011; Moley et al., 2024; Rhind et al., 2022) show that most triathlon injuries occur in running, followed by cycling and swimming, respectively. Bertola et al. (2014), showed in a study conducted in Brazil, that during training and competition 79% of triathletes were injured during running, 16% during cycling and 5% during swimming. Other studies have shown that there is a higher incidence of injury during training than during competition (Egermann et al., 2003; Garós et al., 2024; Hardy et al., 2006). In turn, other studies have found that the more elite the triathlete, the more likely they are to be injured (Burns et al., 2003; Gil-Caselles and Zafra, 2024; Gil-Caselles et al., 2025; Migliorini, 2011; Rogers et al., 2024; Vleck and Garbutt, 1998), so it is very important to control and know the triathlete’s exposure to injury (Gil-Caselles et al., 2023). As a general rule, physical injuries are always associated with a mental health disorder that may influence the emotional and social aspects of the athlete. It would also be essential to approach injury prevention and recovery from a biopsychosocial perspective which considers both the physical and mental aspects of the athlete (Gómez-Correa, 2016).

In the review conducted by Gil-Caselles et al. (2024), a bidirectional relationship between mental health and sports injuries is identified. Furthermore, it has been identified how mental health symptoms and disorders such as anxiety, depression and stress can lead to increased vulnerability to injury in athletes (Rogers et al., 2024), as it is evident that symptoms associated with mental health indicators in athletes influence or precede injury, which negatively affects and prolongs the recovery process (Foo et al., 2023; Schinke et al., 2018). Another important fact to take into account is that women have significantly higher depressive symptomatology than men (Junge and Feddermann-Demont, 2016; Schaal et al., 2011), which can even be twice as high (Reardon et al., 2019). Regarding anxiety and stress levels, scientific evidence shows similar data to the above, confirming a higher prevalence of symptoms in women (Foskett and Longstaff, 2018).

Perfectionism is a multifaceted personality trait, which involves the tendency to set high standards of performance and achievement, both for oneself and others (Hosseini et al., 2023; Stoeber et al., 2017). The multidimensional classification of perfectionism, proposed by Stoeber (2018), distinguishes between perfectionistic concerns and perfectionistic strivings, which allows for a better understanding of how these traits affect sport performance (Gotwals et al., 2012; Stoeber et al., 2017). Furthermore, personal perfectionism, with its external pressures, often shows a negative correlation with psychological wellbeing, contributing to increased stress and anxiety (Damián Núñez et al., 2024).

In the last 10 years, there has been an increase in studies on the psychological factors associated with triathlon at a competitive level. From the sports field, several studies have been carried out that analyse the levels of perfectionism in sports samples (Arenillas and Ruiz-Barquín, 2023; Gil-Caselles et al., 2025; Hernández and Reyes, 2017; Marquiegui and Ruiz-Barquín, 2023), identifying that athletes with a high tendency toward perfectionism have a higher risk of suffering traumatic injuries (De Maria et al., 2024), poorer performance (Nordin-Bates et al., 2024), and negative moods following competitions due to perceived underachievement of goals (Waleriańczyk et al., 2023). In the sport of Triathlon, studies have been carried out (Gil-Caselles et al., 2025; Marquiegui and Ruiz-Barquín, 2023), highlighting the importance of this variable, given its strong relationship with injuries and with the symptomatology of mental health disorders. Triathlons have experienced a notable increase in the number of participants in recent years, a notable example is the 2024 Torremolinos-Andalusia Triathlon World Championship Final in Spain, which registered more than 5,500 athletes from 140 countries, increasing participation by almost 50% compared to the previous edition in Pontevedra. Due to the growth in participation in this sport, the need to understand different aspects has increased, both to improve training processes and participant responses in competition (Landers et al., 2013; Lepers et al., 2013; Schellenberg et al., 2025), which is generating the emergence of new research that has attempted to answer these questions (Gil-Caselles et al., 2023; Ríos-Garit and Pérez-Surita, 2020).

The aim of this study was to examine the relationship between mental health indicators and injury vulnerability in triathletes depending on their level (Youth, Elite and Amateur). Specifically, the objectives were: (i) to know the levels of perfectionism, depression, anxiety and stress, and the mood in triathletes; (ii) To explore the relationship between psychological factors of mental health (perfectionism, depression, anxiety, stress and mood) and injury vulnerability.

## Methods

### Design

This study follows a descriptive-cross-sectional and retrospective design as indicated by Ato et al. (2013). Additionally, it is constituted as a descriptive and correlational study, constituting itself as an empirical study based on the description of probabilistic surveys (in this case, psychological tests; León and Montero, 2015; Montero and León, 2007).

### Participants

The study sample consisted of 172 triathletes, with the majority being men (*n* = 114; 66.27%), with women accounting for 33.72% (*n* = 58). Since no differences were found in the scores by gender, the sample was divided into three categories for the following reasons: (i) Youth: this group includes under-23 athletes who have not yet competed in elite level and are still in phase of physical development and muscle maturation. Research on athletic development, aerobic capacity and strength continue to improve until the mid-20s, justifying their classification as young athletes (Bar-Or and Rowland, 2004); (ii) Elite: this category includes athletes competing in national and international events, regardless of being under 23 years old. They undergo selection processes and high-performance training programs, setting them apart from amateur and developmental athletes (Vaeyens et al., 2009); and (iii) Amateur: these athletes primarily engage in triathlon for intrinsic motivation, personal challenges, and overall physical wellbeing.

The participant’s characteristics by group were: (i) Mean age (Amateur, 40.19 ± 8.35; Youth, 19.50 ± 2.63; and Elite, 30.38 ± 8.77); (ii) Mean week training time (Amateur, 5.63 ± 1.07; Youth, 5.52 ± 1.28; and Elite, 6.58 ± 0.67); and (iii) Mean time spent training daily (Amateur, 2.16 ± 1.66; Youth, 2.38 ± 2.12; and Elite, 3.02 ± 2.00).

A purposive non-probabilistic sampling was carried out, where participants were deliberately selected based on specific relevant characteristics for the study (Reales Chacón et al., 2022).

### Instruments

The assessment instruments used for the study were: 113.

- Questionnaire of personal and sport variables. *Ad hoc* questionnaire to collect sociodemographic data of the athlete.
- History of sports injuries. Questionnaire created *ad hoc*, based on an injury protocol (Olmedilla et al., 2017). It collects the number of sports injuries suffered in the last two seasons and 117 specific data on them.
- The Multidimensional Perfectionism Scale (MPS) by Carrasco et al. (2010) adapted from the original Multidimensional Perfectionism Scale by Frost et al. (1990). It consists of 35 items with statements to which subjects must respond according to their level of agreement/disagreement on a Likert-type response scale ranging from 1 (Strongly Disagree) to 5 (Strongly Agree). The questionnaire is composed of 6 subscales corresponding to dimensions of perfectionism, four of them of first order (Fear of errors ME, Parental influences IP, Expectations of achievement EL and Organization O) and other two of second order (Adaptive or functional perfectionism and maladaptive or dysfunctional perfectionism). The levels of reliability obtained are Cronbach’s *α* for the total scale is *α* = 0.93, and for the four subscales *α* = 0.88 in ME, *α* = 0.90 in PI, *α* = 0.87 in EL and *α* = 127 0.89 in O.
- The Depression, Anxiety and Stress Scale-21 items (DASS-21, Lovibond and Lovibond, 1995) in its version adapted and validated in Spanish (Fonseca-Pedrero et al., 2010). It has been used to measure general symptoms of depression, anxiety and stress. This scale has three subscales: 131 depression, anxiety and stress, each composed of 7 items, for a total of 21 items. In the present study, 132 the reliability levels obtained for the total scale and for the sub-factors are a Cronbach’s *α* of 0.81. In 133 the present study, the pro-rated depression, anxiety and stress variables correspond to the total score divided by 7 (the number of items), and the pro-rated and non-pro-rated data can also be presented.
- Profile of Mood States (POMS, McNair et al., 1971). In its Spanish version adapted and validated by Fuentes et al. (1995). It is a self-report questionnaire to measure mood. The short version was used, with 29 items answered on a Likert-type scale with 5 response options. It includes five dimensions: Tension (*α* = 0.83); Depression (*α* = 0.78); Anger (*α* = 0.85); Vigor (*α* = 0.83); Fatigue (*α* = 0.82). The variable Depression EA refers to mood states, the nomenclature is given to differentiate it from other instruments that analyses the same variable.

### Procedure

In order to carry out this study, the triathletes were contacted (by telephone) and the purpose of the study was explained to them, the objective of the study, as well as the confidentiality of their data, both their answers and the data previously collected (Rosero-Duque and Navas-Martínez, 2024). In addition, they were given instructions on how to complete the questionnaires and were sent the link to the questionnaire via WhatsApp so that they could fill it in online.

The tests were carried out on a voluntary basis, without the use of any type of financial incentive. The study complies with ethical and deontological requirements in accordance with the Declaration of Helsinki (World Medical Association (WMA), 2000; Bošnjak, 2001; Tyebkhan, 2003). All participants signed informed consent and voluntarily participated in the study. Furthermore, the study complies with the Standards of Ethics in Sport and Exercise Science Research (Harriss et al., 2019) and, in turn, all aspects of the Code of Good Research Practice of the universities to which the various co-authors belong have been considered.

In the medical-health field there are many indicators of injury severity (Jiménez, 2020), but in the bibliometric review reviewed there is no indication of a weighted injury severity index such as the TGSP (global time margin in which the athlete is out of training or competitions due to injury in the entire evaluation period carried out).

In this study, the weighted injury severity index variable is transformed as the TGSP (global time margin in which the athlete is out of training or competitions due to injury in the whole evaluation period) and the days in which the athlete is injured are added up according to their severity:

1. Mild. When treatment is required and at least 1 day without training. Value 2.5 average days off work.
2. Moderate. When it requires treatment 6 days or more without training and loss of some competition. Value of 18 days average sick leave.
3. Severe. When it requires 1–3 months of sick leave; sometimes hospitalization and even surgery. Value of 60 days average sick leave.
4. Very serious. When it requires more than 4 months of sick leave; sometimes, it produces a permanent decrease in sporting performance; constant rehabilitation. Value of 135 average days of sick leave. Example: An athlete who has had two moderate injuries in February: 18–18 = 36 days off; an athlete who has had five minor injuries in the 6 months: 2.5 + 2.5 + 2.5 + 2.5 + 2.5 = 12.5 days off.

### Data analysis

The data analysis is presented in tables, combining both descriptive and inferential analysis. Conducted using the statistical technique of bivariate correlations to enhance the understanding of the employed analysis. To assess normality assumptions, the Kolmogorov–Smirnov test was conducted (significance level *p* < 0.05). However, not all variables met the normality criteria. Consequently, a z-score transformation was applied to approximate normality. Despite this adjustment, normality assumptions were still not satisfied, necessitating the use of non-parametric tests. To determine the degree of association between two variables, the Spearman correlation coefficient was used. The significance threshold was set at *p* < 0.05, meaning that correlations with *p*-values below this threshold led to the rejection of the null hypothesis. The strength of correlations was classified according to Schober et al. (2018) as follows: (i) no correlation from 0.00 to 0.01; (ii) very weak correlation from 0.10 to 0.19; (iii) weak correlation from 0.20 to 39; moderate correlation from 0.40 to 0.59; strong correlation from 0.60 to 0.79; and very strong correlation from 0.80 to 1.00. All statistical analyses were conducted using Jamovi statistical software (version 2.4.14 for Windows).

## Results

Regarding the study objective of analyzing the correlation between perfectionism, depression, anxiety, stress, and mood in triathletes with TNL and TGSP, the following statistical analyses were performed.

Table 1 presents the descriptive statistics and bivariate correlations between TNL-z and TGSP-z across three competitive levels (Amateur, Youth, and Elite). It includes mean, median, standard deviation, and interquartile range for each variable, along with the corresponding Spearman’s rho correlation coefficients.

**Table 1:** Descriptive statistics and correlations for study TNL and TGSP.

Variable	Level	*N*	Mean	Median	SD	IQR	Spearman’s rho (TNL–z)	Spearman’s rho (TGSP–z)
TNL–z	Amateur	122	1.65	1.50	1.13	1.00	1.00	0.61***
TGSP–z		122	29.51	18.00	45.06	33.50	0.61***	1.00
TNL–z	Youth	26	2.00	2.00	1.47	2.00	1.00	0.73***
TGSP–z		26	41.60	23.00	41.46	70.38	0.73***	1.00
TNL–z	Elite	25	2.36	2.00	1.11	1.00	1.00	0.41*
TGSP–z		25	87.98	56.50	133.14	115.00	0.41*	1.00

^*^p < 0.05, **p < 0.01, ***p < 0.001. –z = correlation performed with a z score transformation.

A significant positive correlation was observed between TNL and TGSP across all levels: (i) Amateur group, “strong correlation” 2; (ii) Youth group: “strong correlation”; and (iii) Elite group, “moderate correlation”: These findings indicate higher TNL scores were consistently associated with higher TGSP scores, with the strongest correlation observed in the Youth group.

Table 2 explores the relationships between TNL and four psychological factors: achievement expectations, paternal influence, error aversion, and organization across the amateur, youth, and elite athletes.

**Table 2:** Descriptive statistics and correlations for TNL and TGSP with vulnerability variables.

Perfectionism	Level	*N*	Mean	Median	SD	IQR	(TNL–z)		(TGSP–z)	
							r*_p_*	*p*	r*_p_*	*P*
Achievement expectations	Amateur	121	23.88	23.00	7.14	10.00	0.01	0.903	0.071	0.071
	Youth	25	26.52	26.00	7.87	8.00	−0.16	0.443	0.924	0.924
	Elite	25	25.60	25.00	4.50	6.00	−0.30	0.151	0.854	0.854
Paternal influence	Amateur	121	15.36	14.00	6.51	6.00	−0.08	0.390	−0.19	0.043
	Youth	25	18.84	16.00	8.38	14.00	0.04	0.883	0.14	0.492
	Elite	25	16.92	17.00	6.04	4.00	0.14	0.500	−0.16	0.452
Error aversion	Amateur	121	23.08	21.00	8.74	11.00	0.00	0.965	−0.13	0.159
	Youth	25	25.60	24.00	8.15	9.00	0.07	0.736	0.23	0.278
	Elite	25	25.48	26.00	6.93	9.00	0.10	0.638	−0.01	0.979
Organization	Amateur	121	24.02	24.00	4.50	7.00	−0.09	0.343	−0.24	0.007
	Youth	25	23.60	23.00	6.18	10.00	−0.06	0.760	0.01	0.974
	Elite	25	24.36	26.00	5.44	9.00	−0.35	0.085	−0.24	0.247

–z = correlation performed with a z score transformation.

The findings in Table 2 showed: (i) Paternal Influence (Amateur): a statistically significant negative correlation was found between TGSP and Paternal Influence (very weak correlation); and (ii) Organization (Amateur): a significant negative correlation was observed between TGSP and Organization (weak correlation), suggesting that athletes with lower organizational tendencies had higher TGSP scores.

The associations between TNL and TGSP with four perfectionism dimensions: adaptive perfectionism, maladaptive perfectionism, adaptive and maladaptive differential perfectionism, and total perfectionism are reported in Table 3.

**Table 3:** Descriptive statistics and correlations for TNL and TGSP with perfectionism variables.

Perfectionism 4 factor	Level	*N*	Mean	Median	SD	IQR	(TN–z)		(TGSP–z)	
							r*_p_*	*p*	r*_p_*	*p*
4-factor adaptive perfectionism -	Amateur	121	47.91	48.00	9.61	12.00	−0.01	0.874	−0.13	0.159
	Youth	25	50.12	48.00	10.39	12.00	−0.05	0.810	0.23	0.278
	Elite	25	49.96	51.00	8.32	12.00	−0.39	0.052	−0.01	0.979
4-factor maladaptive perfectionism	Amateur	121	38.44	35.00	13.77	16.00	−0.03	0.783	−0.19	0.043
	Youth	25	44.44	43.00	13.74	22.00	0.02	0.912	0.14	0.492
	Elite	25	42.40	39.00	11.19	17.00	0.07	0.726	−0.16	0.452
4-factor adaptive and maladaptive differential perfectionism	Amateur	121	9.48	11.00	11.25	10.00	−0.02	0.805	−0.17	0.071
	Youth	25	5.68	6.00	12.58	14.00	−0.19	0.375	0.02	0.924
	Elite	25	7.56	12.00	12.75	19.00	−0.35	0.085	0.04	0.854
Total perfectionism	Amateur	121	86.35	83.00	20.94	27.00	−0.04	0.665	−0.24	0.007
	Youth	25	94.56	96.00	20.87	33.00	−0.04	0.862	0.01	0.974
	Elite	25	92.36	91.00	15.05	19.00	−0.17	0.420	−0.24	0.247

–z = correlation performed with a z score transformation.

The association performed in Table 3 displayed: (i) Maladaptive Perfectionism (Amateur): a statistically significant negative correlation with TGSP (very weak) suggests that higher levels of maladaptive perfectionism are associated with lower TGSP scores; (ii) Total Perfectionism (Amateur): a weak negative correlation was found between TGSP and Total Perfectionism; (iii) No significant correlations were found for TNL.

Table 4 presents the relationships between TNL and TGSP with three psychological variables: Depression, Anxiety, and Stress.

**Table 4:** Descriptive statistics and correlations for TNL and TGSP with depression, anxiety and stress.

Dass-21	Level	*N*	Mean	Median	SD	IQR	(TNL–z)		(TGSP–z)	
							r*_p_*	*p*	r*_p_*	*p*
Depression	Amateur	121	3.65	2.00	4.11	5.00	0.06	0.545	−0.06	0.497
	Youth	25	5.31	4.50	4.99	6.00	0.39	0.051	0.55	0.004
	Elite	25	3.96	3.00	3.70	6.00	0.48	0.014	0.33	0.102
Anxiety	Amateur	121	2.83	1.00	4.03	3.00	0.14	0.123	0.02	0.849
	Youth	25	5.42	4.00	5.49	6.75	0.06	0.753	0.32	0.110
	Elite	25	2.88	1.50	3.18	3.50	0.09	0.692	−0.08	0.726
Stress	Amateur	121	4.99	3.00	5.42	7.00	0.12	0.206	−0.08	0.373
	Youth	25	6.56	6.00	4.54	7.00	0.41	0.039	0.49	0.012
	Elite	25	6.96	6.00	4.28	6.50	0.08	0.714	0.12	0.589

– z = correlation performed with a z score transformation.

A statistically significant positive correlation was observed in Table 4: (i) Elite athletes: a statistically significant positive correlation was found between TNL and Depression (moderate effect); (ii) Amateur athletes: TGSP and depression showed a significant positive correlation (strong effect); (iii) Youth athletes: stress was moderately correlated with TNL, and a statistically significant moderate correlation was observed between TGSP and Stress. These results suggest that higher levels of depression and stress are positively associated with both TNL and TGSP, particularly among Elite and Youth athletes.

Table 5 examines the relationships between TNL, TGSP and five emotional states: Tension, Anger, Depression, Vigor, and Fatigue.

**Table 5:** Descriptive statistics and correlations for TNL and TGSP and five emotional states.

Poms	Level	*N*	Mean	Median	SD	IQR	(TNL–z)		(TGSP–z)	
							r*_p_*	*p*	r*_p_*	*p*
Tension	Amateur	121	8.45	7.00	5.69	9.00	0.03	0.725	−0.18	0.045
	Youth	25	11.60	12.00	5.30	7.00	0.46	0.020	0.61	0.001
	Elite	25	9.63	1.00	5.09	7.50	−0.07	0.734	0.00	0.987
Anger	Amateur	121	8.19	5.00	6.66	7.00	0.08	0.390	−0.01	0.901
	Youth	25	1.04	9.00	6.56	1.00	0.36	0.074	0.58	0.003
	Elite	25	9.75	1.00	5.90	8.50	0.07	0.729	0.15	0.479
Depression	Amateur	121	2.78	1.00	4.44	3.00	−0.03	0.755	−0.13	0.153
	Youth	25	4.20	3.00	4.05	6.00	0.40	0.048	0.65	<0.001
	Elite	25	4.17	3.50	3.95	6.50	0.30	0.156	0.22	0.304
Vigor	Amateur	121	13.86	15.00	3.57	4.00	−0.14	0.123	−0.14	0.126
	Youth	25	13.40	12.00	3.03	4.00	0.26	0.206	−0.08	0.707
	Elite	25	12.92	14.00	4.03	5.25	−0.56	0.004	−0.51	0.010
Fatigue	Amateur	121	6.10	5.00	4.80	6.00	0.06	0.534	−0.03	0.748
	Youth	25	8.12	8.00	4.92	7.00	0.22	0.281	0.38	0.063
	Elite	25	5.92	5.00	4.53	6.50	0.02	0.936	0.19	0.385

– z = correlation performed with a z score transformation.

Table 5 indicated: (i) Amateur athletes: TGSP and Tension showed a negative correlation (weak effect); (ii) Youth athletes: TNL and Tension (moderate effect) and TGSP and Tension (strong effect) showed significant positive correlations. TNL and Depression (moderate effect) and TGSP and Depression (strong effect) also exhibited positive correlations; and (iii) Elite athletes: A negative correlation was found between TNL and Vigor (moderate effect). TGSP and Vigor also had a moderate negative correlation. These finding indicate that higher levels of tension and depression are linked to greater TNL and TGSP scores, while higher vigor is associated with lower TNL and TGSP scores in Elite athletes.

## Discussion

The aim of this study was to examine the relationship between mental health indicators and injury vulnerability in triathletes depending on their level (youth, elite and amateur). Specifically, the objectives were: (i) To know the levels of perfectionism, depression, anxiety and stress, and the mood in triathletes; (ii) To explore the relationship between psychological factors of mental health (perfectionism, depression, anxiety, stress and mood) and injury vulnerability.

Regarding the first objective, to determine the levels of perfectionism, depression, anxiety, and stress, and the mood of triathletes, the results show that the Organization factor of the Perfectionism Scale of the MPS questionnaire, the three factors of the DASS-21 questionnaire (stress, anxiety, and depression), and the Tension and Anger mood states of the POMS questionnaire present significant relationships, both positive and negative, with NLT and TGLSP. These relationships, although low in magnitude, indicate that they describe temporary or long-term alterations in mental health. Negative or inverse relationships were found between NLT and/or TGLSP and the Organization and Vigor factors, and positive relationships with Stress, Anxiety, Depression, and Anger.

Regarding the second objective, to determine the relationship between the psychological aspects of mental health and vulnerability to injury (Gil-Caselles et al., 2024), the relationship between perfectionism and vulnerability to injury has been studied (De Maria et al., 2024). The results show that athletes with a higher level of maladaptive perfectionism tend to experience higher levels of physical anxiety, characterized by physical or physiological tension, and social anxiety, which manifests as discomfort, avoidance, and discomfort toward other people or places (Taylor et al., 2007). This anxiety, in turn, can increase vulnerability to injury, as it interferes with concentration, technique, and emotional control, key factors in avoiding accidents during sports practice (Fisher, 2024). Similarly, recent studies have shown that perfectionism may also be linked to a higher risk of injury in sports such as American football, track and field, and basketball (Gil-Caselles et al., 2025; Meng et al., 2024; Mesa et al., 2023; Olmedilla et al., 2022a, 2022b), and some of the published studies on perfectionism also state that the higher the level of adaptive perfectionism, the lower the symptoms of anxiety, depression, and stress, and conversely, the higher the levels of maladaptive perfectionism, the higher the levels of these symptoms (Olmedilla et al., 2022a, 2022b). In conclusion, a statistically positive relationship was observed between maladaptive perfectionism and injury likelihood, and an inverse, negative relationship between maladaptive perfectionism and adaptive perfectionism (Gil-Caselles et al., 2023).

Our results are partially consistent with the findings of De Maria et al. (2024), as we found that triathletes with high levels of maladaptive perfectionism reported greater vulnerability to injury, particularly in relation to physical exertion and social anxiety. However, unlike their study, which focused on a general population of athletes, ours focused specifically on triathletes. Stoeber et al. (2017) study with triathletes highlights how psychological factors interact with each other and how perfectionism can have both positive and negative effects, depending on its nature (adaptive or maladaptive) and how athletes manage their expectations and goals. Due to the unique combination of disciplines in triathlons, triathletes face diverse physical and psychological stresses that may differentially influence the relationship between perfectionism and injury (Parsons-Smith et al., 2022; Schellenberg et al., 2025). These differences in the study population may explain some of the discrepancies observed in our results.

All these results are not confirmed in the present study carried out in a cross-sectional manner, given that there are no correlations between the injury indices used (TLN and/or TGLSP) and the scores for adaptive, maladaptive or total perfectionism. However, inverse correlations of low magnitude, but significant, have been found between TGLSP and the Organization factor. That is, no correlations are shown with global scores, but there does seem to be a slight tendency for athletes with lower organization scores to show a slight tendency toward the weighted injury severity index (TGLSP). These correlations can be increased with the control of variables and sample segmentation, such as the analyses.

Regarding the relationship between depression, anxiety and stress, and vulnerability to injury, the results show that the highest scores are obtained in the stress factor, followed by Depression and the lowest in Anxiety. Thanks to the Andersen and Williams model (1988), it is possible to understand how the athlete’s stress response implies an increase in their vulnerability to injury. Since the appearance of these models until today, the scientific literature has provided empirical evidence showing that the athlete’s stress responses and history of stressors have the strongest associations with injury rates (Catalá and Peñacoba, 2020; Ivarsson et al., 2017). In addition, the relevant role of stress has been added to other factors such as anxiety and depression, allowing us to speak of a negative triad of vulnerability to sports injury (Olmedilla et al., 2022a, 2022b). In our study, considering the average scores of the DASS-21 questionnaire, low scores are obtained (below 1 point on a scale of 0 to 3). Authors such as Weinberg and Gould (2010) highlight the psychological benefits of practicing aerobic activities for the prevention of anxiety and depressive states, although this is limited to a sport and health perspective and not a sports performance perspective. From this perspective, and in order to establish an adequate interpretation of the results, it is important to generate specific scales with the sample of triathletes, given that, either due to the previous psychological conditions of the athletes, or due to the psychophysiological adaptations resulting from the training and competition loads in the sport of triathlon, the levels of stress, anxiety and depression are significantly lower than those of the general population. It is therefore confirmed that anxiety and stress emerge as predictive factors of injuries, and if any of these psychological factors are present, they can trigger or exacerbate the others, further increasing the risk of injuries. For example, if an athlete experiences anxiety and does not receive treatment, they could develop symptoms of depression, and in turn, they could increase their risk of injury (Gil-Caselles and Zafra, 2024). Another relevant aspect is that statistically significant weak positive correlations have been obtained between the total number of injuries with the three factors that make up the DASS-21, coinciding with the study by Olmedilla et al. (2009) and Olmedilla et al. (2014) where it was shown that higher levels of anxiety are associated with a greater number of injuries.

On the other hand, no statistically significant correlations are shown between the variable TGSP and the three factors that make up the DASS-21 questionnaire, although in other studies such as Castro Sánchez et al. (2016) on profiles of vulnerability to sports injury, they suggest that the severity of the injury may be key to determining a low or high risk of suffering some type of sports injury. In turn, in the longitudinal study by Kiliç et al. (2018) results were obtained where the most serious injuries have a greater probability of developing symptoms of common mental disorders, in addition to increasing states of anxiety and stress. Regarding the relationship between mood and vulnerability to injury, the results show that the variable anger directly, and vigor inversely, seem to play a significant role in the relationship between psychological factors and injuries. A possible interpretation of the results obtained with the relationship between anger and the number of injuries is associated with emotions of anger and helplessness in the face of the repetition of an injury of greater or lesser severity in a short period of time. Some studies show that athletes with high levels of perfectionism tend to experience negative emotions, such as anger, especially when they fail to achieve their goals or when injuries prevent them from fully participating in their sport (Flett and Hewitt, 2002; Stoeber, 2018).

Moreover, in many studies, the role of the mood state anger has been associated to a greater extent with its role as an enhancer or inhibitor in sports performance (Cox, 2008; Morgan, 1980). According to Morgan (1980), according to the Mental Health Model, the performance profile is high scores in vigor, and low scores in anger, depression, fatigue, confusion and tension. The greater the difference between positive and negative scores, the greater the existence of the so-called “Iceberg Profile.” Already Cox (2008), in a review of studies, indicates how in sports such as karate, the mood state of anger can have an adaptive value in combat sports or in those that involve high levels of energy or aggressiveness. In the study by Parsons-Smith et al. (2022), it was shown that mood states predict sports performance and reflect the state of mental health. In addition, some differences in mood by gender and age group were evident, with men and older triathletes reporting lower tension than women and younger triathletes. It should not be forgotten that longer duration sports are more susceptible to mood fluctuations during the event compared to short duration sports, which reduces the predictive effectiveness of pre-event mood on subsequent performance (Parsons-Smith et al., 2022). In a study with soccer players, athletes who had high scores on the rumination, despair, and magnification subscales of the Pain Catastrophizing Scale (PCS) scored higher on the factors of tension, depression, anger, and fatigue than players with the lowest scores on the same three subscales. Therefore, the appearance of mood states that could be higher in injured triathletes could have an adaptive function from the perspective of being an activating or mobilizing element. However, from the perspective of Clinical and Health Psychology, it is known that high levels of anger could have negative effects on a person’s mental and physical health (González-García and Martinent, 2020; Muñoz-Villena et al., 2020). A key finding based on the low prevalence of the most negative mood profiles, compared to population norms and other sporting samples, is that triathlon participation is associated with a lower risk of mental illness. Therefore, this finding is highly encouraging for those participating in the sport of triathlon (Mawlichanów et al., 2024) as an athlete’s mental illness symptoms become more severe, their performance can be negatively affected, leaving them vulnerable and exposed to more symptoms of common mental disorders (Souter et al., 2018).

## Conclusion

Relationships have been found between total number of injuries (TNL) and total injury severity (TGSP) with the variables considered, especially scores obtained with stress, anxiety and depression with mood states, and negative relationships have been found with the organizational factor of the personality trait perfectionism. This study reported a positive correlation between the variables total number of injuries (TNL) and total injury severity (TGSP). Furthermore, positive results were obtained between the number of injuries and the three factors (stress, anxiety and depression), with stress being the highest. Therefore, although a relationship was observed between mental health indicators and sports injuries in triathletes, further research is required to establish with certainty whether symptoms of any of these indicators directly influence greater vulnerability to injury or slower recovery.

## Limitations of the study

Despite the efforts to recruit a larger sample, it has not been possible, since most triathletes have found it difficult to complete the test battery. On the other hand, within the sample a higher number of men than women has been obtained, and this data can hinder the research since it is important to consider how gender or sports category by sex can affect perceptions of mental health and illness.

In addition, some studies suggest that men have more stigmatizing attitudes toward those with Depression (Cook and Wang, 2010; Wang et al., 2007) and Anxiety (Batterham et al., 2013) compared to women. In turn, men are highly competitive, and it is interesting to know their physical and mental strength in relation to and/or in comparison to women.

### Practical applications

The findings of this study provide knowledge about the vulnerability of triathletes to injury as a consequence of alterations in mental health. It is very important to know which mental health indicators affect triathletes and how they do so, especially in the incidence of injuries and/or in the recovery from them. The fact that a triathlete is more exposed to injury due to the development of one or more mental health indicators (anxiety, depression and stress) makes it interesting and very important to investigate and know its prevalence. All the data obtained should be relevant in the field for professionals in Sports Sciences. It is necessary to know the sport that is practiced, its physical and mental requirements and how the athlete faces its practice and the consequences that may arise from them.

### Future lines of research

Education should address the importance of mental health in the sports field. Not only educating and training, but also raising awareness about the existence of mental health problems and explaining their complexity (Foulkes, 2022). Future research should explore the links between sports injuries and mental health indicators in terms of which are more relevant or which are most developed in triathletes.

Since it is a sport that is constantly growing, it is vital and very important to continue along this line of research, and to expand it by learning about the differences between genders and between competition categories. In addition, future studies should specifically analyze the relationship between levels of adaptive and maladaptive perfectionism in relation to behaviors directly or indirectly related to sports performance, such as, for example, performance limitations such as sports injuries. Triathlon is a very complex and demanding sport on a physical and mental level, and knowledge must continue to be provided so that in the future, thanks to the multidisciplinary work of different specialists, triathletes can be mentally and physically strong.

On the other hand, it is important to consider in future studies the 6-factor model, and not just the 4-factor model, in order to establish possible relationships between perfectionism levels and the injury indices considered (total number of injuries and weighted injury severity index), when evaluating perfectionism using the MPS questionnaire. Although the 4-factor model is configured as an attempt to reorganize the six primary factors of the perfectionism questionnaire, the greater specificity of the 6-factor model could help to further specify the relationships with the injury indicators.

## Data Availability

The raw data supporting the conclusions of this article will be made available by the authors, without undue reservation.
